# Supplementary material for: Data-driven reaction coordinate discovery in overdamped and non-conservative systems: application to optical matter structural isomerization
Source: Nat Commun. 2021 May 5;12:2548. doi: 10.1038/s41467-021-22794-w (PMC8099877; doi:10.1038/s41467-021-22794-w)
Supplement: Supplementary file 1 — Supplementary Information [file 41467_2021_22794_MOESM1_ESM.pdf]

Supplementary Information for Data-Driven Reaction Coordinate Discovery in Overdamped and non-Conservative Systems: Application to Optical Matter Structural Isomerization

Shiqi Chen[1,3], Curtis W. Peterson[1,3], John A. Parker[2,3], Stuart A. Rice[1,3], Andrew L. Ferguson[4,*], and Norbert F. Scherer[1,3,*]

[1] *Department of Chemistry, University of Chicago, Chicago, IL 60637, USA*

[2] *Department of Physics, University of Chicago, Chicago, IL 60637, USA*

[3] *James Franck Institute, University of Chicago, Chicago, IL 60637, USA*

[4] *Pritzker School of Molecular Engineering, University of Chicago, Chicago, IL 60637, USA*

[*] *nfschere@uchicago.edu, andrewferguson@uchicago.edu (orcid.org/0000-0002-8829-9726)*

The supplementary information contains figures that show comparisons between experimental and simulation results and provide supporting details to the figures and discussion of the main text.

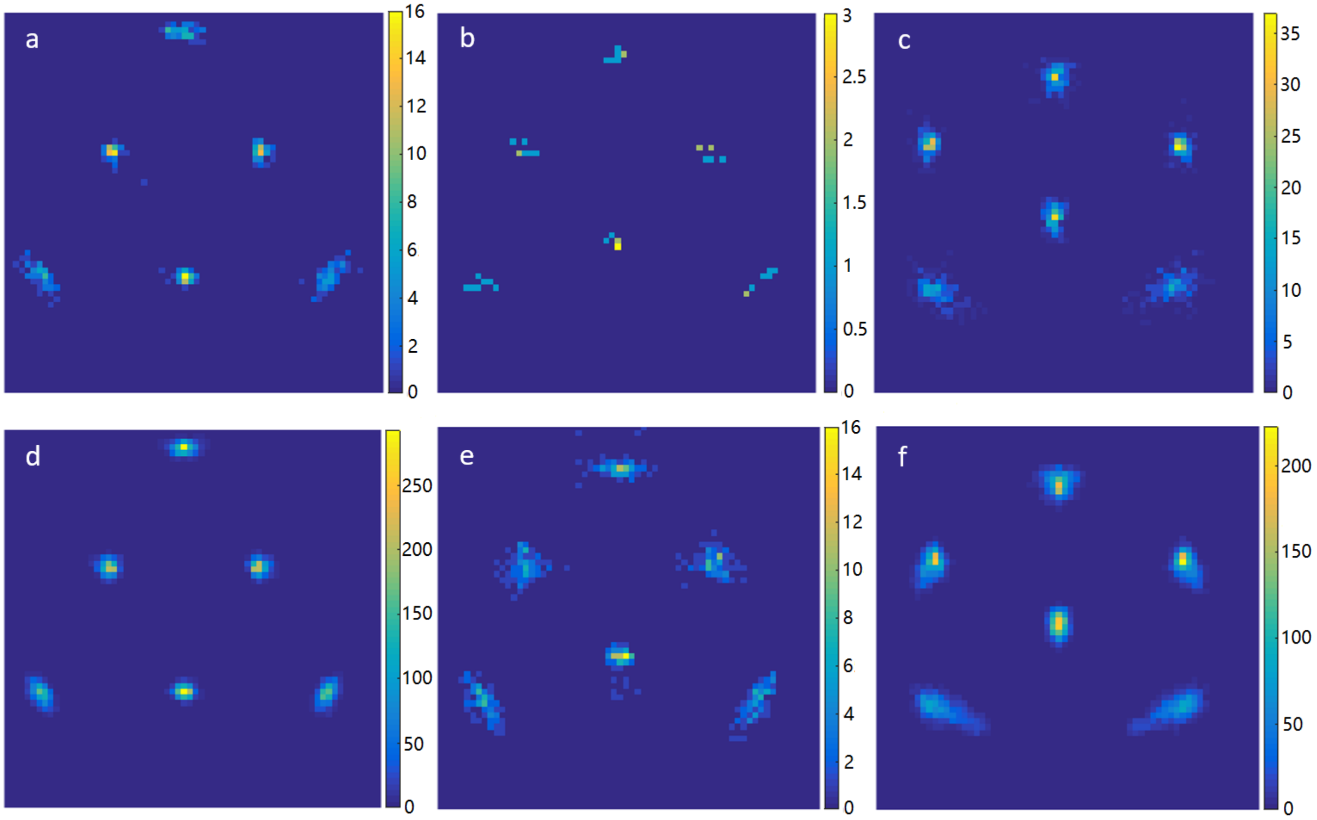

Supplementary Figure 1. Conditional probability distributions of constituent nanoparticles of 6-particle optical matter clusters conditioned on the distance between the two particles on the (vertical) symmetry axis (cf. Fig. 2). The color scheme describes the number of configurations that contains a particle centered at a specific pixel. (a-c) Experimental distributions collected over 1686 configurations at an optical power of 50 mW. (d-f) Simulation result collected 21186 configurations at an optical power of 70 mW.

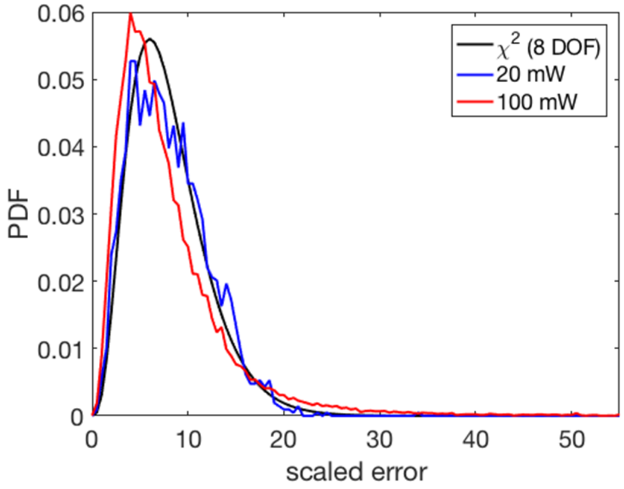

Supplementary Figure 2. Probability density function (PDF) representation of lattice fitting displacement distributions of the 6-particle optical matter system corresponding to the cumulative density functions shown in Figure 3. The lattice fitting displacement is the sum of squares of particle position deviations of a configuration from the stable triangle lattice sites minimized over all possible translations, rotations, and the lattice parameter (i.e., lattice site spacing). The fitting displacement computed from electrodynamics-Langevin dynamics (EDLD) simulation trajectories of fluctuations in the vicinity of the triangle configuration deviates from the 8 degree of freedom χ^2 distribution. The magnitude of the deviation of the PDF fitting displacement distribution from the 8 DOF χ^2 distribution (solid black curve) increases with optical trapping power in simulations conducted over the range 20-100 mW, indicating that the collective motions become more significant (and increase in magnitude) at higher optical powers (i.e. for stronger simulated optical trapping fields).

Fig. 5 of the main text presents a detailed HLDA analysis of a single triangle-to-chevron transition of a 6-particle optical matter cluster as well as statistical analysis of many transitions observed in simulations. Of course, each transition behaves differently, so Supplementary Fig. 3 shows some of the variations for representative transitions. In general, PC mode 3 is dominant for almost all transitions, but different modes can contribute significantly at various times during the structural transition.

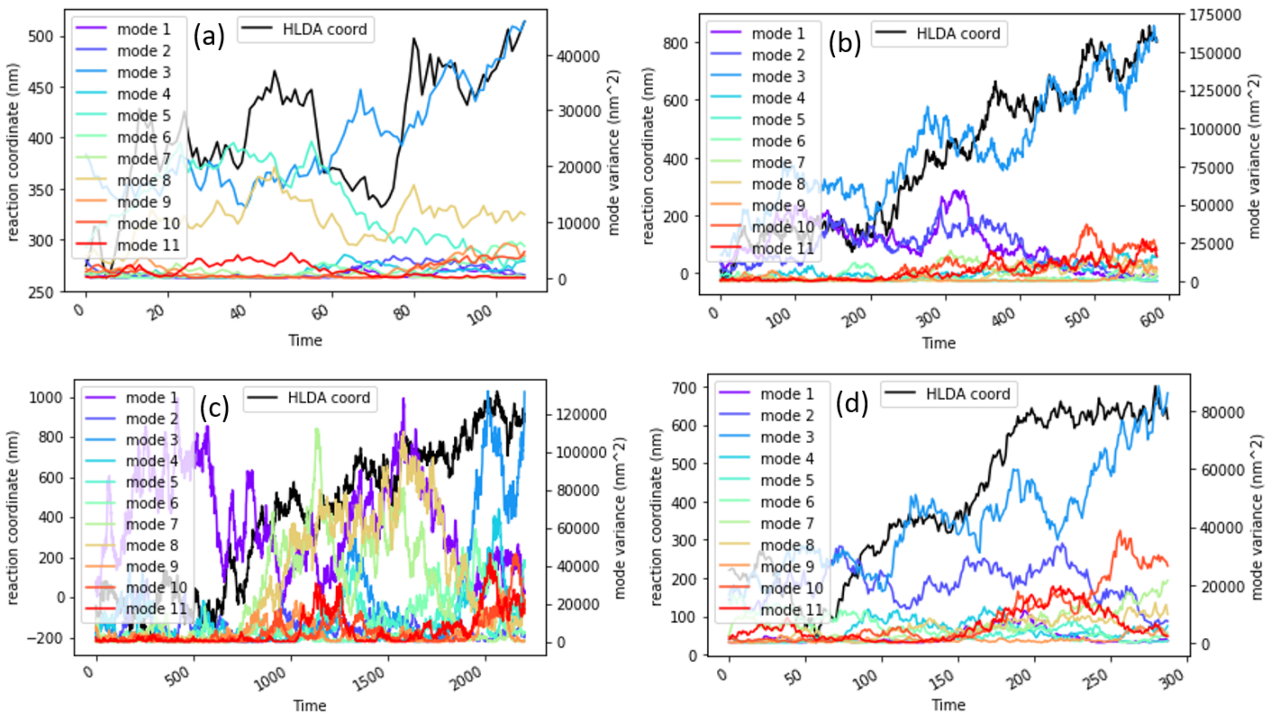

Supplementary Figure 3. Comparison of the HLDA reaction coordinate s_{HLDA} and the variance of particle deviations along each of the 12 collective modes over the course of four different triangle-to-chevron isomerization transitions (cf. Fig. 6a). Some transitions occur quickly like Supplementary Fig. 3a, but some are more prolonged like Supplementary Fig. 3c. However, at the end of the transition, mode 3 always dominates.

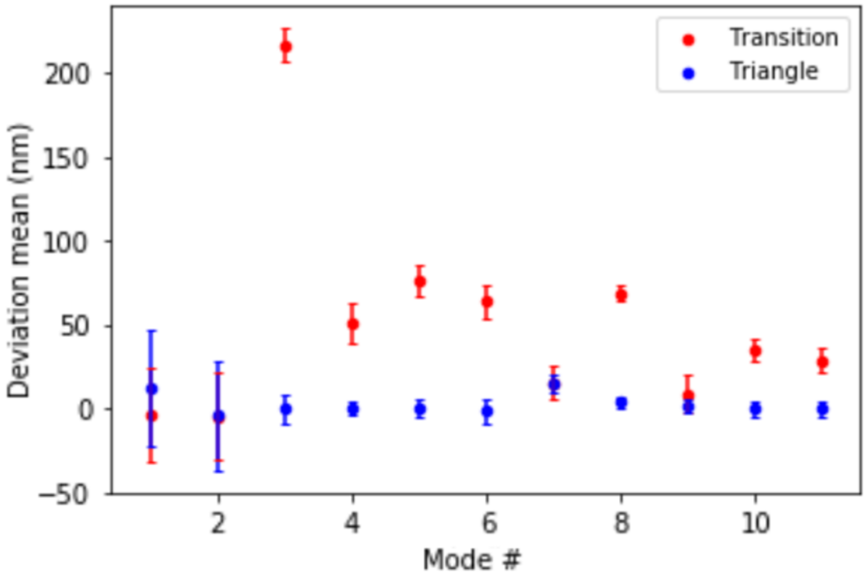

Supplementary Figure 4. The error bars plotted for the mean of the deviations projected on to the 11 modes for triangle and transition configurations plotted in Fig. 6c. The error bars correspond to the standard deviations of the distributions shown in Fig. 6c

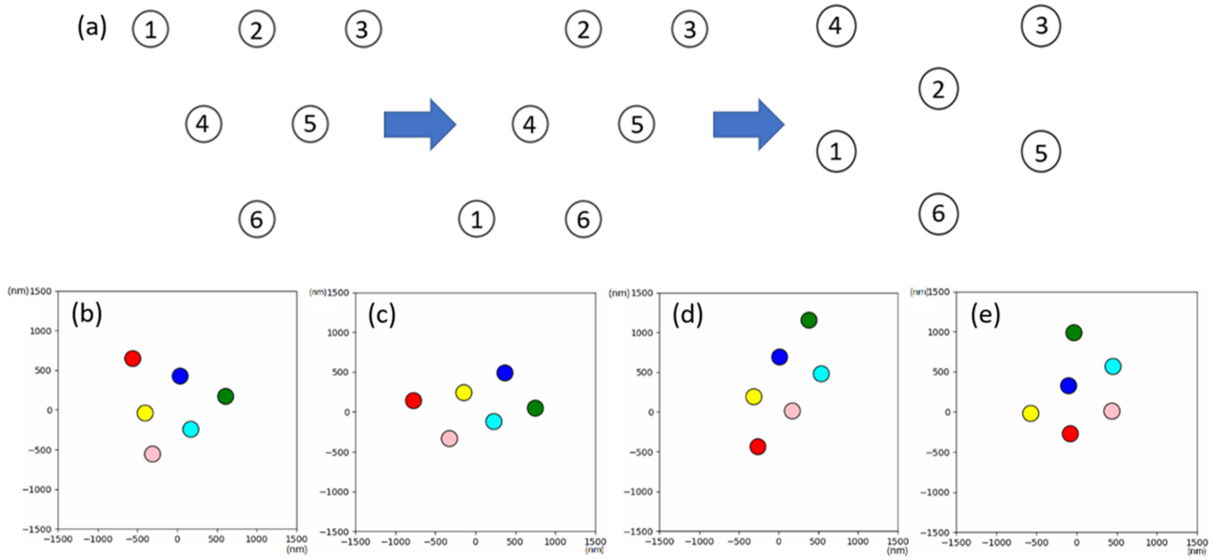

Supplementary Figure 5. An indirect triangle-chevron transition. **a** The transition scheme with labeled particles. **b-c** Initial and final states of first half of transition. **d-e** Initial and final states of second half of transition. In this case, the intermediate OM structure has a parallelogram shape, which is different from the triangle "reactant" and chevron "product".

Supplementary Discussion

Derivation of the relationship between conventional normal modes and statistical collective modes in conservative systems.

Let us consider a system with N identical particles in $\mathbb{R}^d$. A d-dimensional position vector, $\mathbf{r}_k = [r_{k1}, r_{k2}, ..., r_{kd}]$ is assigned to the kth particle. The configuration of the whole system is described by the Nd-dimensional configuration vector:

$$\mathbf{R} = (\mathbf{r}_1, \mathbf{r}_2, ..., \mathbf{r}_N) = [r_{11}, r_{12}, ..., r_{1d}, r_{21}, r_{22}, ..., r_{2d}, .., r_{N1}, r_{N2}, ..., r_{Nd}] \in \mathbb{R}^{Nd} \tag{1}$$

Next, consider a force field acting on this $\mathbb{R}^{Nd}$ configuration space. The force acting on a certain particle not only depends on the position of itself, but also on the positions of other particles. The function $\mathbf{F}$ takes in the position vector of the N particles and produces the forces acting on these N particles. Since the force acting on a particle is a vector in $\mathbb{R}^d$, $\mathbf{F}$ maps the configuration space into the N-particle force space that is also Nd-dimensional.

$$\mathbf{F} : \mathbb{R}^{Nd} \longrightarrow \mathbb{R}^{Nd} \tag{2}$$

The force derivative matrix $\mathbf{K}$ is defined by taking derivatives over all position coordinates of the force function.

$$K_{ip,jq} = \frac{\partial F_{ip}}{\partial r_{jq}} \tag{3}$$

Since the system is conservative, the potential difference between two different configurations is well-defined as the integral of the force field over any path that connects them. We choose a reference configuration $\mathbf{R}^{(0)}$ and define its potential to be zero. Then for any other configuration $\mathbf{R}$, its potential is defined as:

$$V(\mathbf{R}) = \int_{\mathbf{R}}^{\mathbf{R}^{(0)}} \mathbf{F}(\hat{\mathbf{R}}) \cdot d\hat{\mathbf{R}} \tag{4}$$

From the equations above, it is obvious that we can find the force function $\mathbf{F}$ from the potential function by taking negative gradient.

$$\mathbf{F} = -\nabla V \tag{5}$$

Therefore, the force derivative matrix $\mathbf{K}$ is the negative of the Hessian matrix of V.

$$K_{ip,jq} = \frac{\partial F_{ip}}{\partial r_{jq}} = -\frac{\partial^2 V}{\partial r_{ip} \partial r_{jq}} \tag{6}$$

When a configuration is at a local minimum of the potential, its energy is at a local minimum. The system dynamics near the minimum is determined, to second order by

$$V(\mathbf{R}) = V(\mathbf{R}_0) + \nabla V(\mathbf{R}_0) \cdot (\mathbf{R} - \mathbf{R}_0) + \frac{1}{2}(\mathbf{R} - \mathbf{R}_0)^{\mathrm{T}}[\nabla \nabla^{\mathrm{T}} V(\mathbf{R}_0)](\mathbf{R} - \mathbf{R}_0) + o(\|\mathbf{R} - \mathbf{R}_0\|_2^2) \tag{7}$$

Since V is at its local minimum at $\mathbf{R}_0$, the gradient of V at $\mathbf{R}_0$ is zero. Therefore, the first order term vanishes and the second order term dominates. In addition, we can replace the Hessian matrix of V by the negative of force derivative matrix $\mathbf{K}$.

$$V(\mathbf{R}) = V(\mathbf{R}_0) - \frac{1}{2}(\mathbf{R} - \mathbf{R}_0)^{\mathrm{T}} \mathbf{K} (\mathbf{R} - \mathbf{R}_0) + o(\|\mathbf{R} - \mathbf{R}_0\|_2^2) \tag{8}$$

At a non-zero temperature T, the system configuration $\mathbf{R}$ is a random variable that has a probability distribution. The probability density for a configuration $\mathbf{R}$ to appear at a non-zero temperature T follows the Boltzmann distribution:

$$p(\mathbf{R}) \propto e^{-\frac{V(\mathbf{R})}{k_B T}} \tag{9}$$

k_B is the Boltzmann constant. Then,

$$p(\mathbf{R}) \propto e^{-\frac{V(\mathbf{R}) - V(\mathbf{R}_0)}{k_B T}} = e^{\frac{(\mathbf{R} - \mathbf{R}_0)^{\mathrm{T}} \mathbf{K} (\mathbf{R} - \mathbf{R}_0)}{2 k_B T} + o(\|\mathbf{R} - \mathbf{R}_0\|_2^2)} \tag{10}$$

Therefore, the probability distribution of configurations near the stable configuration $\mathbf{R}_0$ is close to a Gaussian distribution. Let the covariance matrix of this Gaussian distribution be Σ. Then according to the distribution function, we will have the following relationship.

$$\mathbf{K}(\mathbf{R}_0)\Sigma = -k_B T \mathbf{I}_{Nd \times Nd} \tag{11}$$

Therefore, any normal mode of the system, which is one of the eigenvectors of the force derivative

matrix $\mathbf{K}$, is also an eigenvector of the covariance matrix Σ. This is because, for any $\mathbf{v}$ that satisfies:

$$\mathbf{K}\mathbf{v} = \lambda \mathbf{v} \tag{12}$$

we have:

$$\Sigma v = k_B T \mathbf{K}^{-1} \mathbf{v} = \frac{k_B T}{\lambda} \mathbf{v} \tag{13}$$

Note that the derivation above cannot be applied to non-conservative systems directly due to reasons stated below. Since the potential is not well-defined in our system, the Hessian matrix should be replaced by taking the first derivative of the external force field, the J matrix, instead of taking the second derivative of the potential. The J-matrix approach is an important method that can be compared to the present work. The J-matrix method leads to discovery of oscillatory solutions[1], which indeed sounds promising in analyzing non-conservative systems, but is limited to underdamped systems. In the overdamped case relevant for optical matter systems, the Langevin equation is first order so that there is no oscillatory solution. In the overdamped OM system, the J-matrix method is still applicable, but since the absence of oscillatory solutions, the application is limited to the analysis of its eigenvectors and eigenvalues. Furthermore, since the J-matrix is asymmetric, its eigenvectors are not orthogonal to each other and its left eigenvectors are different from its right eigenvectors, which induces coupling between the collective modes represented by its eigenvectors. We note that the covariance matrix analyzed using PCA gives orthogonal eigenvectors that make an orthogonal basis much easier to manipulate.

Supplementary Methods

Derivation of the analytical expressions of optimal lattice fitting parameters given lattice assignment. $\mathbf{1}$ is the vector of all ones. Given $\mathbf{p}, \mathbf{q} \in \mathbb{C}^N$ (configuration coordinates), solve the optimal parameter set $\{a^*, \theta^*, p_0^*\}$ for:

$$Err^* = \left[\min_{a\in\mathbb{R}^+, p_0\in\mathbb{C}, \theta\in[0,2\pi)} \left| e^{i\theta}(\mathbf{p} + p_0\mathbf{1}) - a\mathbf{q} \right|^2 \right]^{\frac{1}{2}} \tag{14}$$

We first optimize the translation, fixing $\theta = 0$ and $a = 1$.

$$\begin{aligned}
Err^2\big|_{\theta=0,a=1} &= |(\mathbf{p} + p_0\mathbf{1}) - \mathbf{q}|^2 = [(\mathbf{p} - \mathbf{q}) + p_0\mathbf{1}]^{\mathrm{H}}[(\mathbf{p} - \mathbf{q}) + p_0\mathbf{1}] \\
&= (\mathbf{p} - \mathbf{q})^{\mathrm{H}}(\mathbf{p} - \mathbf{q}) + (p_0\mathbf{1})^{\mathrm{H}}(p_0\mathbf{1}) + (p_0\mathbf{1})^{\mathrm{H}}(\mathbf{p} - \mathbf{q}) + (\mathbf{p} - \mathbf{q})^{\mathrm{H}}(p_0\mathbf{1}) \\
&= (\mathbf{p} - \mathbf{q})^{\mathrm{H}}(\mathbf{p} - \mathbf{q}) + Np_0\overline{p_0} + \overline{p_0}[\mathbf{1}^{\mathrm{H}}(\mathbf{p} - \mathbf{q})] + p_0\overline{[\mathbf{1}^{\mathrm{H}}(\mathbf{p} - \mathbf{q})]} \\
&= |\mathbf{p} - \mathbf{q}|^2 - \frac{1}{N}\left|(\mathbf{p} - \mathbf{q})^{\mathrm{H}}\mathbf{1}\right|^2 \\
&+ N\left\{ p_0\overline{p_0} + \overline{p_0}\left[\frac{\mathbf{1}^{\mathrm{H}}}{N}(\mathbf{p} - \mathbf{q})\right] + p_0\overline{\left[\frac{\mathbf{1}^{\mathrm{H}}}{N}(\mathbf{p} - \mathbf{q})\right]} + \left[\frac{\mathbf{1}^{\mathrm{H}}}{N}(\mathbf{p} - \mathbf{q})\right]\overline{\left[\frac{\mathbf{1}^{\mathrm{H}}}{N}(\mathbf{p} - \mathbf{q})\right]} \right\} \\
&= |\mathbf{p} - \mathbf{q}|^2 - \frac{1}{N}\left|(\mathbf{p} - \mathbf{q})^{\mathrm{H}}\mathbf{1}\right|^2 + N\left|p_0 + \frac{\mathbf{1}^{\mathrm{H}}}{N}(\mathbf{p} - \mathbf{q})\right|^2
\end{aligned} \tag{15}$$

In order to minimize the expression above, one sets the last term of the expression above to zero, which means:

$$p_0^* = \frac{\mathbf{1}^{\mathrm{H}}}{N}(\mathbf{q} - \mathbf{p}) \tag{16}$$

It should be noticed that the expression of p_0^* is actually the difference between the centers of mass of the two configurations, $\mathbf{p}$ and $\mathbf{q}$. In addition, after $\mathbf{p}$ and $\mathbf{q}$ are translated to let both centers of mass overlap with the origin, p_0^* will stay unchanged no matter how p and q are rotated or linearly scaled. Therefore, after the choice of p_0^*, further optimization of θ and a does not affect p_0^*, so that their optimization can be carried out successively and independently.

Next, we optimize the rotation and lattice parameter. Let $p' = p + p_0^*\mathbf{1}$, we have:

$$Err^* = \left[\min_{a\in\mathbb{R}^+, \theta\in[0,2\pi)} \left| e^{i\theta}\mathbf{p}' - a\mathbf{q} \right|^2 \right]^{\frac{1}{2}} \tag{17}$$

$$
\begin{aligned}
Err^2\big|_{p_0=p_0^*} &= \left|e^{i\theta}\mathbf{p}' - a\mathbf{q}\right|^2 = \left|\mathbf{p}' - ae^{-i\theta}\mathbf{q}\right|^2 = \left|\mathbf{p}'\right|^2 + a^2\left|\mathbf{p}'\right|^2 - 2a\mathrm{Re}(\mathrm{e}^{\mathrm{i}\theta}\mathbf{q}^{\mathrm{H}}\mathbf{p}') \\
&= a^2\left|\mathbf{q}\right|^2 - 2(a\left|\mathbf{q}\right|)\left[\frac{\mathrm{Re}(\mathrm{e}^{\mathrm{i}\theta}\mathbf{q}^{\mathrm{H}}\mathbf{p}')}{\left|\mathbf{q}\right|}\right] + \left[\frac{\mathrm{Re}(\mathrm{e}^{\mathrm{i}\theta}\mathbf{q}^{\mathrm{H}}\mathbf{p}')}{\left|\mathbf{q}\right|}\right]^2 - \left[\frac{\mathrm{Re}(\mathrm{e}^{\mathrm{i}\theta}\mathbf{q}^{\mathrm{H}}\mathbf{p}')}{\left|\mathbf{q}\right|}\right]^2 + \left|\mathbf{p}'\right|^2 \\
&= \left|\mathbf{q}\right|^2\left[a - \frac{\mathrm{Re}(\mathrm{e}^{\mathrm{i}\theta}\mathbf{q}^{\mathrm{H}}\mathbf{p}')}{\left|\mathbf{q}\right|^2}\right]^2 + \left\{\left|\mathbf{p}'\right|^2 - \left[\frac{\mathrm{Re}(\mathrm{e}^{\mathrm{i}\theta}\mathbf{q}^{\mathrm{H}}\mathbf{p}')}{\left|\mathbf{q}\right|}\right]^2\right\}
\end{aligned}
\tag{18}
$$

In order to minimize Err, the first term in the equation above should be set to zero, and the second term should be as small as possible, meaning that the real part of $e^{i\theta}\mathbf{q}^{\mathrm{H}}\mathbf{p}'$ is maximized to be $\left|\mathbf{q}^{\mathrm{H}}\mathbf{p}'\right|$. Therefore:

$$
e^{i\theta^*} = e^{-i\arg(\mathbf{q}^{\mathrm{H}}\mathbf{p}')} = \frac{(\mathbf{p}')^{\mathrm{H}}\mathbf{q}}{\left|\mathbf{q}^{\mathrm{H}}\mathbf{p}'\right|} \tag{19}
$$

$$
a^* = \frac{\mathrm{Re}(\mathrm{e}^{\mathrm{i}\theta}\mathbf{q}^{\mathrm{H}}\mathbf{p}')}{\left|\mathbf{q}\right|^2} = \frac{\left|\mathbf{q}^{\mathrm{H}}\mathbf{p}'\right|}{\left|\mathbf{q}\right|^2} \tag{20}
$$

This is the optimal rotation angle and the optimal lattice parameter.

Supplementary References

[1] Chattoraj, J., Gendelman, O., Ciamarra, M. P., Procaccia, I. Oscillatory Instabilities in Frictional Granular Matter. *Phys. Rev. Lett.* **123**, 098003 (2019).
