# Supplementary material for: Data-driven reaction coordinate discovery in overdamped and non-conservative systems: application to optical matter structural isomerization
Source: Nat Commun. 2021 May 5;12:2548. doi: 10.1038/s41467-021-22794-w (PMC8099877; doi:10.1038/s41467-021-22794-w)
Supplement: Supplementary file 3 — Description of Additional Supplementary Files [file 41467_2021_22794_MOESM3_ESM.pdf]

Description of Additional Supplementary Files

File Name: Supplementary Movie 1
Description: Example video of 6-particle optical matter cluster motions
